# Dental Fees

**Published:** 1862-06

**Authors:** J. Taft


### DENTAL REGISTER OF THE WEST.

Vol. XVI.] JUNE, 1862. [No. 6.

### Original Essays and Communications.

### DENTAL FEES.

BY J. TAFT.

This is a subject about which there should be a clear and definite understanding. Two things constitute the basis of a fee or remuneration, or in other words, two elements should always enter into it. The one is value, good, or advantage, accruing to the person for whom the service is rendered; the other is the labor, time, skill and means of, and responsibility assumed by, the person rendering the service. So far as the first of these elements is concerned, with the physician or dentist, a fee can not be brought to a par value; for no patient ever pays his dentist the full value of good, efficient operations upon his teeth, nor does the man restored to health pay the physician an equivalent for his restoration. So far, then, as the dentist is concerned, his fee must be *regulated*, chiefly, by the time, labor, skill and means employed in any given case. Of course, in the employment of these, it must be presumed that the patient has received that which is valuable to him, and though a fee is not regulated according to this, yet without it, there would be no basis for a fee, for no one could demand a fee for services that resulted in no good to the person served. Though there could be no ground for a fee without the latter, yet the former, viz.: labor, time, skill, etc. of the operator, is that which mainly regulates fees. These particulars should all be taken into consideration. "Time is money" is a common proverb; and no one has a right to use the time of another for his own advantage without compensation; and much less should any one, in addition to this, use the labor and skill of another without reward. Those who properly appreciate their teeth will always seek the highest skill, for their preservation, and will not hesitate to amply reward the labor and skill employed for that purpose. By such a course only can the highest attainments be made.

It is not possible, neither would it be right, to make a uniform scale of fees for dental operations, for there is a great variation in the ability of different operators, and indeed of the same operator at different times, and in different places. Fees can not be regulated by the edict of any professional association; there is a large proportion of the profession that anything of this kind could not reach. If any one desires larger fees, the only legitimate way to attain that end is to make the operations such that the patient will feel that the demand is just, and then by intelligent patients it will be freely accorded.

It is a very frequent complaint in the profession that the fees usually charged are too small; if this is applied to the work as it *should* be done, it is perhaps correct, but if to the work as it *is* done, it is perhaps not correct. The probability is that all cheap operators charge as much as they think their work is worth, and as much as it is worth. We accord to all such the right to set the value upon their own operations, and nothing more is claimed for others. If any one thinks his fillings are worth only one dollar each, we are willing to admit that that is all they are worth; while if another thinks his are worth three or five dollars each, if he is a man of integrity, we are bound to receive his estimate as the true one, and all intelligent patients will accede to it.

There are some persons, many indeed, who can not perceive or be made to understand that there is any material difference in operations upon the teeth ; they assume to believe that all are about alike well performed, and that there can not be so much margin for the difference in fees as we find. Such persons pretend to think in this matter as they do not in any other. Every body knows full well that there is a great variety in almost everything ; in all the products of nature we find this variety ; and to perhaps a greater extent do we find it in all the productions of man. Price and value in every thing is governed by this principle of variety, at least it is one of the governing influences. This principle is applicable to the operations of the dentist as truly as to anything else. The " good, bad and indifferent" exist here as elsewhere.

The query is sometimes raised, Should the fees for any given operation be the same for all classes of persons? Most surely not. The fees for dental services should be uniform for those who have the ability to pay. Those who have not should be regarded according to their ability, and no oppression wrought. It is not just to deprive those of limited means of the benefits of good dental operations, neither is it right to impose upon such persons inefficient operations, do something merely for the sake of a fee ; but when anything is done, though it should be for a half fee, or without any fee, it should be done so as to accomplish the object sought ; neither should an operation be performed for a fraction of the usual fee, and the patient left to feel that he is a beneficiary to the extent of the remission, for he has paid as much, according to his ability, as he of ample means, who pays the ordinary fee.

There is another kind of reward that is by no means of small estimation, and one that cheers the operator in many cases as much or more than anything else ; and that is the appreciation of, and the value set upon that which is done, by an intelligent patient. This kind of remuneration is of a far higher character than, and far different from, pecuniary reward ; it is an honor that a low, mean or sordid spirit can not appreciate. That part of a fee such an one never gets. He who has no higher motives, in the performance of his operations, than the money reward which he is to receive, should never be permitted to put an instrument in the mouth. And that patient who will always insist upon having the greatest amount of services (and usually regardless of the character) for the smallest amount of *money* should go toothless, and no operator of integrity and efficiency will desire to come in contact with such an one, professionally.

The cases referred to above, of those of limited means, constitute exceptions, that do not at all conflict with the principles given in the former part of this paper, upon which fees should be based; these principles devolve upon the assumption of ability.

Presuming the operator to be just, he should be kept as nearly untrammelled as possible in the regulation of his fee. If he is cramped and annoyed by the cupidity and dunning propensity of his patient, the character of his operations will most probably, whether he wills it or not, be brought down to a degree corresponding to the contracted reward; if it is not so, he works against his feelings, and against his convictions of right.

From these observations, we think it deducible that one true and efficient method of elevating the dental profession, in the practice at least, is to perform always the most perfect operations possible, and then have the moral courage to demand a just fee; and on the other hand, the best course for the patient is to demand the best possible operations, and then be willing to remunerate for them. By this course *all* will be great gainers, and by the opposite course *all* will suffer loss.

